# Airborne Distributed Position and Orientation System Transfer Alignment Method Based on Fiber Bragg Grating

**DOI:** 10.3390/s20072120

**Published:** 2020-04-09

**Authors:** Wen Ye, Bin Gu, Yun Wang

**Affiliations:** 1 Division of Mechanics and Acoustic Metrology, National Institute of Metrology, Beijing 100029, China; wenye@buaa.edu.cn; 2 China Academy of Electronics and Information Technology, Beijing 100041, China; 3 Prospective Technology Research Department, SAIC Group, Shanghai 200041, China; wangyun@buaa.edu.cn

**Keywords:** distributed position and orientation system, transfer alignment, fiber bragg grating, wing deformation measurement

## Abstract

With the demand for high resolution remote sensing, load array technology has gradually become an effective measure to improve imaging resolution. However, the external flow and internal engine vibration disturbance may lead to the flexible deformation of wings. The traditional rigid baseline error compensation method cannot solve the problem of serious coupling movement error caused by flexible deformation. To address the problem, a transfer alignment model based on fiber Bragg grating for distributed position and orientation system is proposed in this paper. Firstly, based on the multidimensional requirements of flexible deformation information, the layout scheme of fiber Bragg grating was designed, then the continuous strain in the wing surface was obtained after the quadratic fitting of strain measured by fiber Bragg gratings, and the deformation displacement and angle are calculated. Thirdly, flexible deformation compensation for distributed position and orientation system based on fiber Bragg grating was studied. The state equation including position error, velocity error, misalignment angle, and inertial device error was established. The position and attitude information compensated by the flexible lever arm was used as the quantitative measurement. The filtering estimation improved the measurement accuracy of the slave inertial navigation systems. At last, the experiment was carried out and showed that the accuracy of the transfer alignment has been improved significantly.

## 1. Introduction

With the development of the flight platform technology, it is possible to realize simultaneous observation of multi-remote sensing devices on the same platform using multiple observation windows, such as SAR, visible light camera, imaging spectrometer, and laser radar, which can operate at the same time [1,2,3]. Because the observation windows are distributed in different positions of the non-rigid platform, the orientation stability control mode of each remote sensing device is different, and each remote sensing device needs to use Position and Orientation System (POS), in which the Inertial Measurement Unit (IMU) is fixed in different positions together with the load. At the same time, the imaging radar based on the distributed multi-subarray antenna structure, such as the long baseline InSAR and the large array antenna SAR, is developed from single antenna to multi antenna to achieve three-dimensional stereoscopic imaging [4,5]. However, as the performance improves, the more array the element is, the more complex the baseline distribution is, and the more significant the flexible baseline is; the signal processing needs to use POS data information at the subarray level and requires the distributed POS (DPOS) [6,7]. DPOS is composed of a high-precision master IMU, a navigation computer, a global positioning system, and a number of low-precision sub-IMUs. IMU is installed at the different nodes of the carrier, and transfer alignment of each sub-IMU is carried out using the high-precision navigation information obtained from the main POS, which can provide accurate position and orientation information for all the phase points of distributed SAR and other kinds of loads, and achieve high-precision imaging [8].

**Figure 1:**
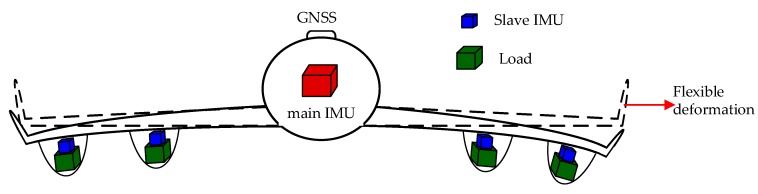
Flexural deformation diagram.

The subarray antenna is often installed at different wing nodes, which is limited by volume weight and even cost and conducts distributed motion measurement often using MEMS-IMU, and its precision is far lower than that of the main system. When the array antenna SAR is three-dimensional imaging, not only is the motion compensation required at each node, but also the required precision of the baseline length between nodes is very high [9]. At present, most of the technical means are accomplished by mathematical modeling or filtering, for example, the second-order Markov process is used to describe the deformation process, in which the parameters are often selected through experience, and the aerodynamics model is used to analyze the wing model [10,11]; the process is very complex, and the wing material, vibration frequency and wing length should be considered. The model based on CKF is used, which features poor anti-interference performance [12,13]. Moreover, the mathematical modeling method has strong pertinence and poor applicability, and it is difficult to achieve precise modeling [14,15]. At the same time, as the following Figure 1 shows, the more nodes, the more the multistage time-varying lever arm error caused by flexible deformation cannot be ignored. The law of deformation between nodes is untraceable and the requirement for the model is harsh [16]. The traditional way of modeling has been difficult to guarantee the measurement precision.

The measurement of deflection deformation based on the inertial navigation system could realize the full dimensional information measurement of the position, velocity, attitude, acceleration, and angular velocity of the sub-nodes, which especially realizes the high-precision measurement of the attitude. However, the error of the inertial navigation system is accumulated with time and it is difficult to achieve the sub-millimeter accuracy measurements of baseline vectors that solely relied on the inertial measurements. At the same time, the fiber Bragg grating (FBG) had small volume and light quality, which has become an important means of monitoring the flexible structural parts in the fields of aerospace large buildings and medical devices, while it is widely used in the internal stress, strain, temperature measurement, and detection of structural fracture and deformation [17]. Various countries in the world are actively developing the deformation measurement technology based on FBG and continue to increase investment in fiber optic sensing development and application of fiber optic sensing. In 1980s, NASA used multiple light emitters to alternate the projection of the central control unit of the engine cabin to analyze the deformation of the wing, and it has been successfully applied to multiple aircraft wing measurements such as YF-12, F-8, and KC-135 [18,19]. In 2010, the Dryden Flight Center of NASA used FBG to measure the flexible deformation of the wing, and calculated the deformation and displacement of the wing by piecewise linear fitting. The optimal relative error was 1% [20]. In 2012, the team further adopted the improved piecewise nonlinear fitting method to reduce the relative error of the displacement to 0.3% [21]. In 2015, NASA carried out a fiber Bragg grating (FBG) deformation measurement experiment on a simulated wing to achieve three-dimensional deformation displacement measurement and two-dimensional angle measurement [22,23]. However, the application of FBG in DPOS has not been reported publicly at home and abroad.

In summary, the deformation measurement based on FBG and the inertial measurement had their own advantages, which are complementary in flexible baseline measurements for array antenna SAR and InSAR. Therefore, this paper used FBG to obtain the three-dimensional deformation between the main node and the sub nodes, which includes the flexural deformation displacement vector and the deflection angles between the main system and the sub-nodes, thus the high-precision measurement information is obtained, and at the same time, the transfer alignment model based on FBG is established. In addition, the according experiment is designed and implemented for wing deformation measurement.

## 2. Composition and Principle of Distributed Pos Based on FBG

**Figure 2:**
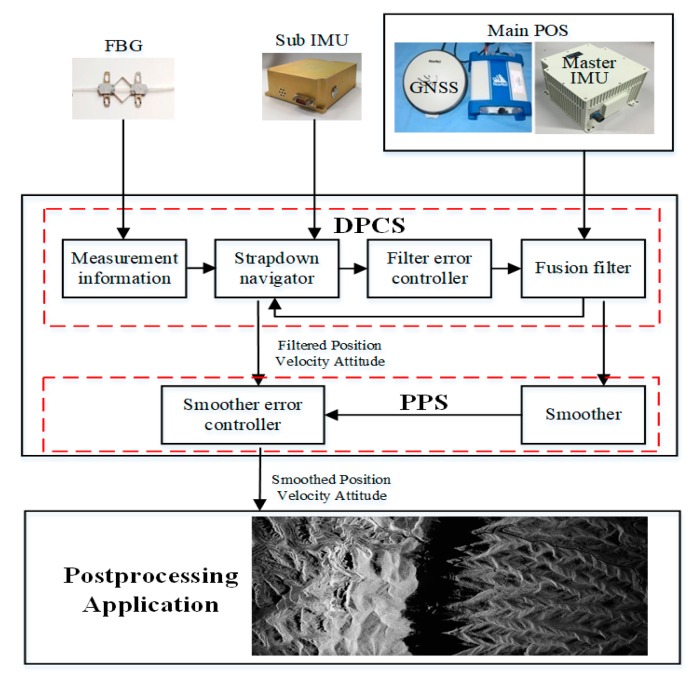
The components and principle of DPOS base on FBG.

The measurement system consists of a high precision main POS, several sub IMUs, a distributed POS computer system (DPCS), a post-processing software (PPS), and multiple fiber Bragg grating sensors. The main POS is generally assembled on the middle of the plane or in the cabin, while the sub IMUs are assembled on or close to every imaging sensor symmetrically distributed along the wings. DPCS is a multifunctional computer system and mainly completes the IMU data sampling and storage, data synchronization, and real-time fusion computation, which includes SINS/GNSS integration for main POS, transfer alignment algorithm for sub IMUs, and communication with other systems. Multiple fiber Bragg grating sensors can provide measurement information about mechanical distortion, which can improve the accuracy of transfer alignment. The motion parameters of DPOS can be calculated in real-time by DPCS or in post-processing by PPS. The PPS is available to calculate a complex model of massive computation without a time limit and can further improve the precision of DPOS. As a result, DPOS can successively provide high-precision position, velocity, and attitude information to further improve imaging accuracy and resolution for multi-task remote sensing sensors. The operation principle of DPOS is shown in Figure 2.

## 3. Deformation Measurement of Fiber Bragg Grating

The FBG measured the flexure deformation displacement vector that was generated by the flexible baseline and the deflection angle around the three axes between the main system and the sub-nodes, that is, the bending variable around the *Y*-axis, the *Z*-axis and the torsion variables around the *X*-axis.

### 3.1. Calculation of Bending Deformation of Y and Z Axis

**Figure 3:**
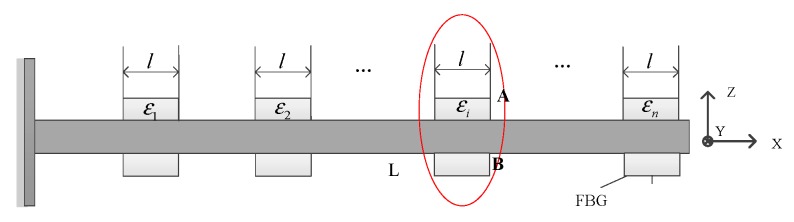
Schematic diagram of the deformation of FBG sensor.

Several fiber Bragg grating sensors are pasted on the wing surface, while assuming that the *i*th pair of FBG sensor is measured as $\varepsilon_{A}$ and $\varepsilon_{B}$, as Figure 3 shows. First, the strain decoupling of each FBG sensor is measured and the linear strain component $\varepsilon_{i}$ that is generated by the bending deformation is separated. Then, FBG sensors are divided into *n* long segments on the wing surface, while each segment could calculate the curvature of the center point by strain. The curvature values at each point are obtained by linear interpolation from the curvature values of the n discrete points. Finally, the deformation angle and the line displacement vector are solved by the geometric relationship.

The sensitive strain of the *i*th pair FBG sensor is symmetric and stickup

(1) $$\{\begin{array}{l} \varepsilon_{A}=\varepsilon_{Fx}+\varepsilon_{Mx}+\varepsilon_{My}+\varepsilon_{Mz}\\ \varepsilon_{B}=\varepsilon_{Fx}+\varepsilon_{Mx}-\varepsilon_{My}+\varepsilon_{Mz} \end{array}$$

In the formula, the linear strain caused by the *X*-axis tensile force, $\varepsilon_{Mx}$, $\varepsilon_{My}$, and $\varepsilon_{Mz}$ is the linear strain caused by the *X*, *Y*, and *Z*-axis bending, respectively. Then the line strain produced by the *Y*-axis bending could be calculated as:

(2) $$\varepsilon_{\mathrm{My}}=\frac{\varepsilon_{A}-\varepsilon_{B}}{2}$$

**Figure 4:**
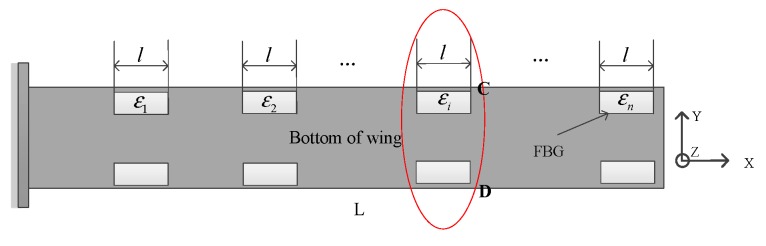
Schematic diagram of deformation measurement of FBG sensor on the lower surface.

In the same way, the strain modeling calculated by the FBG is used to calculate the *X*-*Y* plane displacement vector and the deformation angle along the *Z*-axis; the decoupling of the line strain caused by the pure *Z*-axis bending is as follows in Figure 4:

The susceptibility strain of the FBG sensor symmetrically pasted by the *i*th pair of lower surface is:

(3) $$\{\begin{array}{l} \varepsilon_{C}=\varepsilon_{\mathrm{Fx}}+\varepsilon_{\mathrm{Mx}}-\varepsilon_{\mathrm{My}}-\varepsilon_{\mathrm{Mz}}\\ \varepsilon_{D}=\varepsilon_{\mathrm{Fx}}+\varepsilon_{\mathrm{Mx}}-\varepsilon_{\mathrm{My}}+\varepsilon_{\mathrm{Mz}} \end{array}$$

Then the line strain produced by the *Z*-axis bending could be calculated as

(4) $$\varepsilon_{\mathrm{Mz}}=\frac{\varepsilon_{C}-\varepsilon_{D}}{2}$$

**Figure 5:**
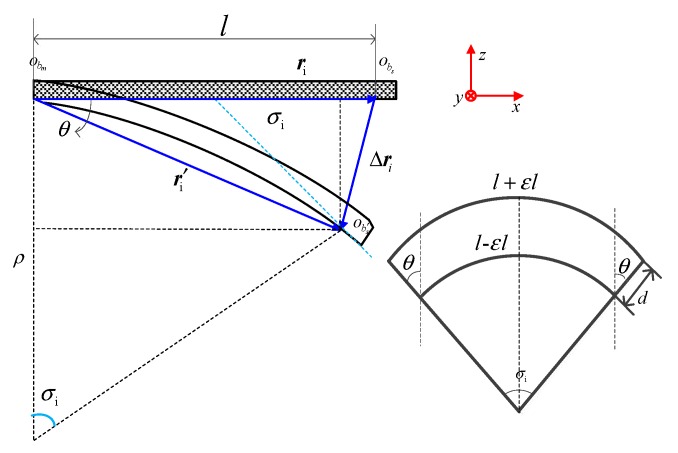
Schematic diagram of the micro arc after the bending deformation of the beam.

The small arc segments after the wing bending deformation are shown in the Figure 5. $\sigma_{i}$ is the relative angle of the cross section of the small arc section, while $\varepsilon_{i}$ is the line strain measured by the FBG sensor. ρ is the radius of curvature corresponding to the small arc segment formed after the deformation of the neutral layer, d is the diameter of the beam, and $r_{i}$ and ${r^{\prime }}_{i}$ are the vector before and after the deformation.

Available from geometric relationships:

(5) $$\theta =\frac{\left(l+\varepsilon_{i}l\right)-\left(l-\varepsilon_{i}l\right)}{2d}=\frac{\varepsilon_{i}l}{d}$$

So that

(6) $$\sigma_{i}=2\theta =\frac{2\varepsilon_{i}l}{d}$$

(7) $$\rho_{i}=\frac{l-\varepsilon_{i}l}{2\varepsilon_{i}l}d=\frac{d}{2\varepsilon_{i}}-\frac{d}{2}$$

According to the stress and strain analysis of the wing, the strain of the wing root is the largest and the tip of the wing is the smallest. It can be known from Formula (7) that the strain is inversely proportional to the radius of curvature obtained by the modeling calculation, that is, when the root strain of the wing tends to zero, the radius of curvature tends to infinity. When there is a small error in the strain measured by the fiber Bragg grating sensor, the radius of curvature would produce a large error, and the displacement vector calculated by the radius of curvature would also have a large error. Since the strain at the tip of the wing is small, the deformation angle is small, and it is assumed that the length before and after the baseline deformation is unchanged, that is $\left|r_{i}\right|\approx \left|{r^{\prime }}_{i}\right|=l$. As shown in Figure 5, according to the geometric relationship $\theta =\sigma_{i}/2$, the displacement vector $r_{xz}^{}$ of *X*-*Z* plane and the deformation angle $\sigma_{y}^{}$ along the direction of *Y* axis in the node coordinate system, the displacement vector $r_{xy}^{}$ of the *X*-*Y* plane and the direction of deformation angle $\sigma_{z}^{}$ along the direction of the *Z* axis in the nodal coordinate system could be expressed as:

(8) $$\left\{ \begin{array}{l} \Delta r_{xz}^{}=\{\begin{array}{cc} -\left[l-\left(\frac{d}{\varepsilon_{A}-\varepsilon_{B}}-\frac{d}{2}\right)\sin\sigma_{y}^{}\right]{\vec{e}}_{x}-\left[\left(\frac{d}{\varepsilon_{A}-\varepsilon_{B}}-\frac{d}{2}\right)(1-\cos\sigma_{y}^{})\right]{\vec{e}}_{y} & \varepsilon_{My}\geq \varepsilon_{y0}\\ -(l-l\cos\frac{\sigma_{y}}{2}){\vec{e}}_{x}-(l\sin\frac{\sigma_{y}}{2}){\vec{e}}_{y} & \varepsilon_{My}<\varepsilon_{y0} \end{array}\\ \sigma^{}{}_{y}=\frac{l(\varepsilon_{A}-\varepsilon_{B})}{d}\\ \Delta r_{xy}^{}=\{\begin{array}{cc} -\left[l-\left(\frac{d}{\varepsilon_{C}-\varepsilon_{D}}-\frac{d}{2}\right)\sin\sigma_{z}^{}\right]{\vec{e}}_{x}-\left[\left(\frac{d}{\varepsilon_{C}-\varepsilon_{D}}-\frac{d}{2}\right)(1-\cos\sigma_{z}^{})\right]{\vec{e}}_{y} & \varepsilon_{Mz}\geq \varepsilon_{z0}\\ -(l-l\cos\frac{\sigma_{z}}{2}){\vec{e}}_{x}-(l\sin\frac{\sigma_{z}}{2}){\vec{e}}_{y} & \varepsilon_{Mz}<\varepsilon_{z0} \end{array}\\ \sigma^{}{}_{z}=\frac{l(\varepsilon_{C}-\varepsilon_{D})}{d} \end{array}\right.$$

### 3.2. Calculation of Torsional Deformation for X-Axis

**Figure 6:**
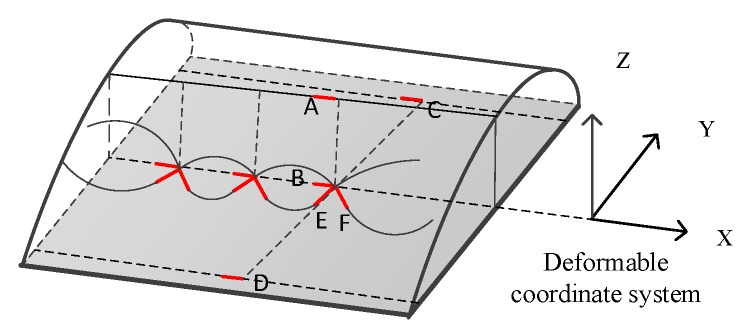
Schematic diagram of fiber grating shear strain measurement.

FBG sensor is only sensitive to the line strain in one direction, so the shear strain generated by the wing under the measurement of the torsional moment could only be obtained by modeling. Figure 6 showed that the lower surface of the wing is pasted with a FBG *B* at 0 degrees, and the measured value of the FBG *E*, *F* with symmetric paste measured at 45° is $\varepsilon_{E}$, $\varepsilon_{F}$.

(9) $$\{\begin{array}{l} \varepsilon_{E}={\varepsilon^{\prime }}_{\mathrm{Fx}}+{\varepsilon^{\prime }}_{\mathrm{Fy}}+{\varepsilon^{\prime }}_{Mx}-{\varepsilon^{\prime }}_{My}+{\varepsilon^{\prime }}_{Mz}\\ \varepsilon_{F}={\varepsilon^{\prime }}_{\mathrm{Fx}}+{\varepsilon^{\prime }}_{\mathrm{Fy}}-{\varepsilon^{\prime }}_{Mx}-{\varepsilon^{\prime }}_{My}-{\varepsilon^{\prime }}_{Mz} \end{array}$$

In the formula, the oblique 45° linear strain caused by the *X*-axis tensile force, ${\varepsilon^{\prime }}_{\mathrm{Mx}}$, ${\varepsilon^{\prime }}_{\mathrm{My}}$, ${\varepsilon^{\prime }}_{\mathrm{Mz}}$ is the oblique 45° linear strain that is caused by the *X*, *Y*, and *Z*-axis bending, respectively. The line strain angle 45° generated at the measuring point under *X*-axis torsion could be calculated as:

(10) $${\varepsilon^{\prime }}_{Mx}=\frac{\varepsilon_{E}-\varepsilon_{F}}{2}-{\varepsilon^{\prime }}_{Mz}$$

According to the geometric relationship of the FBG installation, it could be concluded that there is a relationship between the sensitive line strain of FBG installed in the direction of oblique 45°and the FBG installed at 0°.

(11) $${\varepsilon^{\prime }}_{Mz}=\frac{\varepsilon_{Mz}}{\cos\frac{\pi }{4}}=\frac{\varepsilon_{C}-\varepsilon_{D}}{\sqrt{2}}$$

Substitution of Formula (17) into (16), which could obtain:

(12) $${\varepsilon^{\prime }}_{Mx}=\frac{\varepsilon_{E}-\varepsilon_{F}}{2}-\frac{\varepsilon_{C}-\varepsilon_{D}}{\sqrt{2}}$$

**Figure 7:**
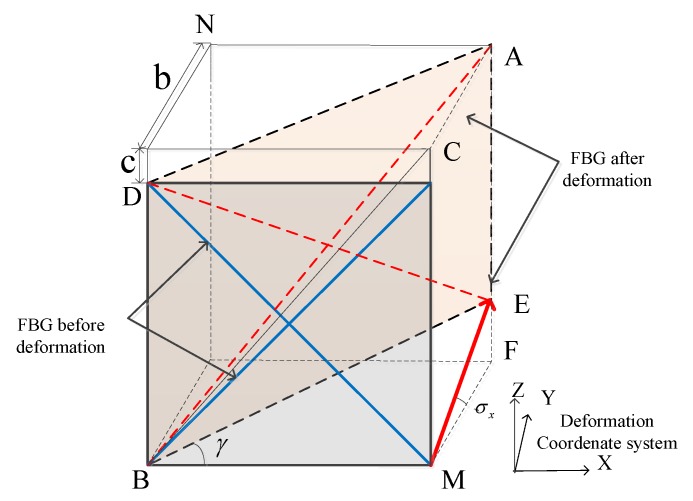
Schematic diagram of fiber grating deformation.

Modeling and analysis of FBG sensors before and after deformation are shown in Figure 7:

In the diagram, the deformation angle $\sigma_{x}$ is the shear strain. The length of the FBG sensor before deformation l is calculated, and the geometry of the plane ADBE is extracted as follows:

The geometric relationship could be obtained:

(13) $$\cos\alpha =\frac{{\left(2l\cos\frac{\pi }{4}\right)}^{2}+{\left(l+\varepsilon l\right)}^{2}-{\left(l-\varepsilon l\right)}^{2}}{2\times 2l\cos\frac{\pi }{4}\times \left(l+\varepsilon l\right)}$$

(14) $$\cos\alpha =\frac{c+l\cos\frac{\pi }{4}}{\left(l+\varepsilon l\right)}$$

It could be solved by Formulas (13) and (14):

(15) $$c=\sqrt{2}\varepsilon l$$

**Figure 8:**
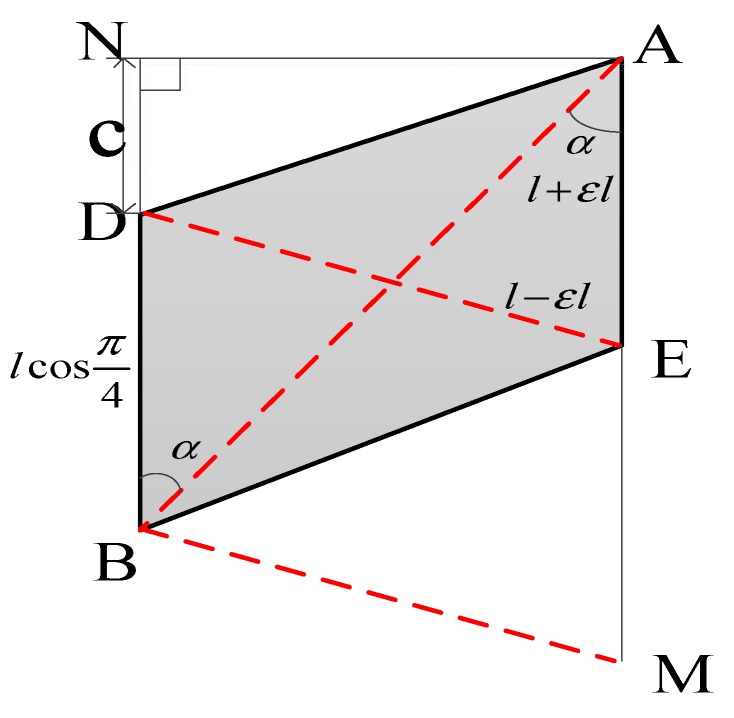
Schematic diagram of fiber Bragg gratings.

From Figure 8, it could be calculated:

(16) $$b=l\sqrt{\left(4\varepsilon -\varepsilon^{2}\right)}$$

When the ε value is very small, $\varepsilon^{2}$ is high-order small quantity, which could be approximated to 0, then the deformation angle $\sigma_{x}$ and displacement vector could be calculated as:

(17) $$\sigma_{x}=arc\tan\left(\sqrt{\frac{\varepsilon }{2}}\right)$$

(18) $$\Delta r_{yz}=\sqrt{2}\varepsilon l{\vec{e}}_{z}+2l\sqrt{\varepsilon }{\vec{e}}_{y}$$

Substituting Equation (15) into Equations (17) and (18), the deformation angle $\sigma_{x}$ and displacement vector $\Delta r_{yz}$ could be expressed as:

(19) $$\left\{ \begin{array}{l} \Delta r_{yz}=l\left[\frac{\varepsilon_{E}-\varepsilon_{F}}{\sqrt{2}}-\left(\varepsilon_{C}-\varepsilon_{D}\right)\right]{\vec{e}}_{z}+2l\sqrt{\frac{\varepsilon_{E}-\varepsilon_{F}}{2}-\frac{\varepsilon_{C}-\varepsilon_{D}}{\sqrt{2}}}{\vec{e}}_{y}\\ \sigma_{x}=arc\tan\left(\sqrt{\frac{\varepsilon_{E}-\varepsilon_{F}}{4}-\frac{\varepsilon_{C}-\varepsilon_{D}}{2\sqrt{2}}}\right) \end{array}\right.$$

## 4. Transfer Alignment Model

### 4.1. Error Equation

The navigation accuracy of the strap-down inertial navigation system is influenced by various error sources, which include the self-error of the inertial sensor, the quantization error of the inertial sensor, the initial condition error, the data truncation error, and various interference errors. In this section, from the mechanism of error propagation, the velocity, position and attitude error equations are derived, and the general error model of strap-down inertial navigation system is also given.

#### 4.1.1. Velocity Error Equation

The velocity errors equation is described as follows:

(20) $$\{\begin{array}{l} \begin{array}{ll} \delta {\dot{V}}_{E} & =f_{N}\phi_{U}-f_{U}\phi_{N}+(\frac{V_{N}\tan L-V_{U}}{R_{N}+H})\delta V_{E}+(2\omega_{\mathrm{ie}}\sin L+\frac{V_{E}\tan L}{R_{N}+H})\delta V_{N}\\ & +(2\omega_{\mathrm{ie}}V_{N}\cos L+\frac{V_{E}V_{N}\sec^{2}L}{R_{N}+H}+2\omega_{\mathrm{ie}}V_{U}\sin L)\delta L-\\ & (2\omega_{\mathrm{ie}}\cos L+\frac{V_{E}}{R_{N}+H})\delta V_{U}+\frac{V_{E}V_{U}-V_{E}V_{N}\tan L}{{\left(R_{N}+H\right)}^{2}}\delta H+\nabla_{E} \end{array}\\ \begin{array}{ll} \delta {\dot{V}}_{N} & =f_{U}\phi_{E}-f_{E}\phi_{U}-2(\omega_{\mathrm{ie}}\sin L+\frac{V_{E}\tan L}{R_{N}+H})\delta V_{E}-\\ & \frac{V_{U}\delta V_{N}}{R_{M}+H}-\frac{V_{N}\delta V_{U}}{R_{M}+H}-(2\omega_{\mathrm{ie}}\cos L+\frac{V_{E}\sec^{2}L}{R_{N}+H})V_{E}\delta L+\\ & \frac{V_{N}V_{U}+V_{E}V_{E}\tan L}{{\left(R_{N}+H\right)}^{2}}\delta H+\nabla_{N} \end{array}\\ \begin{array}{ll} \delta {\dot{V}}_{U} & =-f_{N}\phi_{E}+f_{E}\phi_{N}+2(\omega_{\mathrm{ie}}\cos L+\frac{V_{E}}{R_{N}+H})\delta V_{E}+\\ & 2\frac{V_{N}\delta V_{N}}{R_{M}+H}-2\omega_{\mathrm{ie}}V_{E}\sin L\delta L-\frac{V_{E}V_{E}+V_{N}V_{N}}{{\left(R_{N}+H\right)}^{2}}\delta H+\nabla_{U} \end{array} \end{array}$$

where $\phi^{n}$ is the misalignment angles; $f_{}^{n}$ is the specific force vector with respect to the navigation frame; $\omega_{\mathrm{ie}}^{n}$ is the projection of the Earth’s rotation angular rate in the n-series; $\omega_{\mathrm{en}}^{n}$ is the angular rate of the n-frame relative to the e-frame in the n-frame; $\delta V^{n}$ is velocity error with respect to the n-frame; $V^{n}$ is the velocity in the n-frame; $\delta \omega_{\mathrm{ie}}^{n}$ is the angular velocity error of e-frame with respect to i-frame in the n-frame; $\delta \omega_{\mathrm{en}}^{n}$ is the angular velocity error of n-frame with respect to e-frame in the n-frame; $C_{b}^{n}$ is the attitude transformation matrix of the b-frame to the n-frame; and $\nabla^{b}$ is the constant bias of accelerometers.

#### 4.1.2. Position Error Equation

The position error equation of the location differential equation is derived from the longitude, the position error equation is:

(21) $$\{\begin{array}{l} \delta \dot{L}=\frac{\delta V_{N}}{R_{M}+H}-\frac{V_{N}}{{\left(R_{M}+H\right)}^{2}}\delta H\\ \delta \dot{\lambda }=\frac{\sec L}{R_{N}+H}\delta V_{E}+\frac{V_{E}\sec L\tan L}{R_{N}+H}\delta L-\frac{V_{E}\sec L}{{\left(R_{N}+H\right)}^{2}}\delta H\\ \delta \dot{H}=\delta V_{U} \end{array}$$

#### 4.1.3. Attitude Error Equation

The attitude error angle is defined as follows:

(22) $$\{\begin{array}{l} \begin{array}{ll} {\dot{\phi }}_{E} & =-\frac{\delta V_{N}}{R_{M}+H}+(\omega_{\mathrm{ie}}\sin L+\frac{V_{E}\tan L}{R_{N}+H})\phi_{N}-\\ & (\omega_{\mathrm{ie}}\cos L+\frac{V_{E}}{R_{N}+H})\phi_{U}+\frac{V_{N}}{{\left(R_{M}+H\right)}^{2}}\delta H+\varepsilon_{E} \end{array}\\ \begin{array}{ll} {\dot{\phi }}_{N} & =\frac{\delta V_{E}}{R_{N}+H}-\omega_{\mathrm{ie}}\sin L\delta L-(\omega_{\mathrm{ie}}\sin L+\frac{V_{E}\tan L}{R_{N}+H})\phi_{E}-\\ & \frac{V_{N}}{R_{M}+H}\phi_{U}-\frac{V_{E}}{{\left(R_{N}+H\right)}^{2}}\delta H+\varepsilon_{N} \end{array}\\ \begin{array}{ll} {\dot{\phi }}_{U} & =\frac{\tan L\delta V_{E}}{R_{N}+H}+(\omega_{\mathrm{ie}}\cos L+\frac{V_{E}\sec^{2}L}{R_{N}+H})\delta L+\\ & (\omega_{\mathrm{ie}}\cos L+\frac{V_{E}}{R_{N}+H})\phi_{E}+\frac{V_{N}\phi_{N}}{R_{M}+H}-\frac{V_{E}\tan L\delta H}{{\left(R_{N}+H\right)}^{2}}+\varepsilon_{U} \end{array} \end{array}$$

#### 4.1.4. Inertial Device Error Equation

The inertial sensors error is approximated as a random constant and white noise, and the random constant could be described by the following differential equations:

(23) $$\begin{array}{l} \begin{array}{ccc} {\dot{\varepsilon }}_{x}=0, & {\dot{\varepsilon }}_{y}=0, & {\dot{\varepsilon }}_{z}=0 \end{array}\\ \begin{array}{ccc} {\dot{\nabla }}_{x}=0, & {\dot{\nabla }}_{y}=0, & {\dot{\nabla }}_{z}=0 \end{array} \end{array}$$

where $\varepsilon_{x}$, $\varepsilon_{y}$, $\varepsilon_{z}$ and $\nabla_{x}$, $\nabla_{y}$, $\nabla_{z}$ are respectively the constant drift of the gyros and the constant bias of the accelerometers.

### 4.2. Modeling of Distributed POS Transfer Alignment System Based on Fiber Bragg Grating

#### 4.2.1. Establishment of System State Equation

Based on the analysis of the transfer alignment error model, the fifteen dimensional state variables of the transfer alignment system model are selected:

(24) $$\begin{array}{ll} X=[ & \phi_{x}\phi_{y}\phi_{z}\delta V_{x}\delta V_{y}\delta V_{z}\delta L\delta \lambda \delta h\\ & \varepsilon_{x}\varepsilon_{y}\varepsilon_{z}\nabla_{x}\nabla_{y}\begin{array}{c} \nabla_{z} \end{array}] \end{array}$$

Among them: $\phi =[\begin{array}{ccc} \phi_{x} & \phi_{y} & \phi_{z} \end{array}]$ is the platform misalignment angle of the subsystem; $\delta V={\left[\begin{array}{ccc} \delta V_{x} & \delta V_{y} & \delta V_{z} \end{array}\right]}^{T}$ is the velocity error of the sub-IMU; δL, δλ, δh is the latitude, longitude and altitude error of the subsystem respectively, while $\varepsilon ={\left[\begin{array}{ccc} \varepsilon_{x} & \varepsilon_{y} & \varepsilon_{z} \end{array}\right]}^{T}$ is the drift of the gyro, and $\nabla =[\begin{array}{ccc} \nabla_{x} & \nabla_{y} & \nabla_{z} \end{array}]$ is the accelerometer bias. Establish the transfer alignment state equation as follows:

(25) $$\dot{X}=FX+GW$$

Among them, F is the state transition matrix, G is the system noise matrix, and W is the expression of the sum of the zero-mean Gaussian white noise: $W={\left[\begin{array}{cccccc} w_{\varepsilon x} & w_{\varepsilon y} & w_{\varepsilon z} & w_{\nabla x} & w_{\nabla y} & w_{\nabla z} \end{array}\;w_{\varphi }\;w_{\gamma }\;w_{rx}\;w_{ry}\right]}^{T}$.

#### 4.2.2. Establishment of Measurement Equation

The wing deformation displacement $\Delta r={\left[\Delta r_{x}^{m},\Delta r_{y}^{m},\Delta r_{z}^{m}\right]}^{T}$ and flexible deformation angle $\sigma ={\left[\sigma_{x}^{m},\sigma_{y}^{m},\sigma_{z}^{m}\right]}^{T}$, the position of main node and attitude measurement information are corrected, and the higher-precision measurement of transfer alignment $Z={\left[\begin{array}{cccccc} \delta \psi & \delta \theta & \delta \gamma & \delta L & \delta \lambda & \delta h \end{array}\right]}^{T}$ is obtained.

Since the position and attitude measurement information of the main POS could be obtained by using the fiber grating correction, the position and attitude matching method could be used to establish the measurement equation of the sub-IMU transmission alignment:

(26) Z=HX+v

In the equation, v is the measurement noise, $H=\left[\begin{array}{ccc} H_{11} & 0_{3\times 3} & 0_{3\times 9}\\ 0_{3\times 3} & I_{3\times 3} & 0_{3\times 9} \end{array}\right]$ is the measurement matrix, and its specific expression is as follows:

(27) $$H_{11}=\left[\begin{array}{ccc} \frac{C_{b_{m}}^{n}(1,2)C_{b_{m}}^{n}(3,2)}{{\left[C_{b_{m}}^{n}(1,2)\right]}^{2}+{\left[C_{b_{m}}^{n}(2,2)\right]}^{2}} & 0 & -1\\ -\frac{C_{b_{m}}^{n}(2,2)}{{\sqrt{1-[C_{b_{m}}^{n}(3,2)]}}^{2}} & \frac{C_{b_{m}}^{n}(1,2)}{\sqrt{1-{\left[C_{b_{m}}^{n}(3,2)\right]}^{2}}} & 0\\ \frac{C_{b_{m}}^{n}(2,1)C_{b_{m}}^{n}(3,3)-C_{b_{m}}^{n}(3,1)C_{b_{m}}^{n}(2,3)}{{\left[C_{b_{m}}^{n}(3,3)\right]}^{2}+{\left[C_{b_{m}}^{n}(3,1)\right]}^{2}} & \frac{C_{b_{m}}^{n}(3,1)C_{b_{m}}^{n}(1,3)-C_{b_{m}}^{n}(1,1)C_{b_{m}}^{n}(3,3)}{{\left[C_{b_{m}}^{n}(3,3)\right]}^{2}+{\left[C_{b_{m}}^{n}(3,1)\right]}^{2}} & 0 \end{array}\right]$$

(1) Position measurement information correction

The two level flexible lever arms between the main child nodes included a fixed lever arm and a flexible deformation. Then the position correction formula for the transfer alignment is:

(28) $$\left[\begin{array}{c} \delta L\\ \delta \lambda \\ \delta h \end{array}\right]=\left[\begin{array}{c} L_{m}\\ \lambda_{m}\\ h_{m} \end{array}\right]-\left[\begin{array}{c} L\\ \lambda \\ h \end{array}\right]-\left[\begin{array}{ccc} 1/\left(R_{M}+h_{h}\right) & 0 & 0\\ 0 & \sec L_{m}/\left(R_{M}+h_{h}\right) & 0\\ 0 & 0 & 1 \end{array}\right]C_{b_{m}}^{n}\left[\begin{array}{c} r_{x0}+\Delta r_{x}^{m}\\ r_{y0}+\Delta r_{y}^{m}\\ r_{z0}+\Delta r_{z}^{m} \end{array}\right]$$

where L, λ and h respectively represent the latitude, longitude and altitude before the sub-IMU transfer alignment, $R_{m}$ and $R_{n}$ respectively represent the main meridian circle and the principal radius of curvature of the circle, and $C_{b_{m}}^{n}$ is the attitude matrix of main POS.

(2) Attitude measurement information correction

The attitude error angle between the main POS and the sub-IMU $\mu =[\mu_{x},\mu_{y},\mu_{z}{]}^{T}$ included two parts of the fixed installation error angle $\rho ={\left[\rho_{x},\rho_{y},\rho_{z}\right]}^{T}$ and the flexible deformation angle $\sigma ={\left[\sigma_{x},\sigma_{y},\sigma_{z}\right]}^{T}$, in which the fixed installation error angle could be obtained by the laser total station calibration after the system installation, and the flexible deformation angle could be measured by the FBG sensor.

The attitude relationship between the main POS and the sub-IMU is as follows:

(29) $$C_{b}^{n\prime }=(I_{3\times 3}-\phi \times )C_{b_{m}}^{n}(I_{3\times 3}+\mu \times )$$

where μ=ρ+σ, ϕ× and μ× respectively represent the skew symmetric matrix composed of the misalignment angle ϕ and the error angle μ.

The above formula is developed to eliminate the second-order small amount, and the approximate value is taken:

(30) $$C_{b}^{n\prime }=C_{b_{m}}^{n}+C_{b_{m}}^{n}(\mu \times )-(\phi \times )C_{b_{m}}^{n}$$

Remember $\delta \psi^{\prime }=\psi -\psi_{m}$, $\delta \theta^{\prime }=\theta -\theta_{m}$, $\delta \gamma^{\prime }=\gamma -\gamma_{m}$, which could obtain:

In the formula, $C_{b_{m}}^{n}(i,j)$ represents the element of the *i*th row and *j*th column of the main POS pose matrix. The two sides are expanded according to the Taylor series while the second order and above are ignored.

(31) $$\begin{array}{ll} \delta \psi \prime & =\frac{C_{b_{m}}^{n}(1,2)C_{b_{m}}^{n}(3,2)}{{\left[C_{b_{m}}^{n}(1,2)\right]}^{2}+{\left[C_{b_{m}}^{n}(2,2)\right]}^{2}}\phi_{E}+\frac{C_{b_{m}}^{n}(2,2)C_{b_{m}}^{n}(3,2)}{{\left[C_{b_{m}}^{n}(1,2)\right]}^{2}+{\left[C_{b_{m}}^{n}(2,2)\right]}^{2}}\phi_{N}-\phi_{U}+\\ & \frac{C_{b_{m}}^{n}(1,2)C_{b_{m}}^{n}(2,3)-C_{b_{m}}^{n}(1,3)C_{b_{m}}^{n}(2,2)}{{\left[C_{b_{m}}^{n}(1,2)\right]}^{2}+{\left[C_{b_{m}}^{n}(2,2)\right]}^{2}}\mu_{x}+\frac{C_{b_{m}}^{n}(1,1)C_{b_{m}}^{n}(2,2)-C_{b_{m}}^{n}(1,3)C_{b_{m}}^{n}(2,2)}{{\left[C_{b_{m}}^{n}(1,2)\right]}^{2}+{\left[C_{b_{m}}^{n}(2,2)\right]}^{2}}\mu_{z} \end{array}$$

(32) $$\begin{array}{ll} \delta \theta \prime & =-\frac{C_{b_{m}}^{n}(2,2)}{\sqrt{1-{\left[C_{b_{m}}^{n}(3,2)\right]}^{2}}}\phi_{E}+\frac{C_{b_{m}}^{n}(1,2)}{\sqrt{1-{\left[C_{b_{m}}^{n}(3,2)\right]}^{2}}}\phi_{N}+\\ & \frac{C_{b_{m}}^{n}(3,3)}{\sqrt{1-{\left[C_{b_{m}}^{n}(3,2)\right]}^{2}}}\mu_{x}-\frac{C_{b_{m}}^{n}(1,3)}{\sqrt{1-{\left[C_{b_{m}}^{n}(3,2)\right]}^{2}}}\mu_{z} \end{array}$$

(33) $$\begin{array}{ll} \delta \gamma \prime & =-\frac{C_{b_{m}}^{n}(2,1)C_{b_{m}}^{n}(3,3)-C_{b_{m}}^{n}(3,1)C_{b_{m}}^{n}(2,3)}{{\left[C_{b_{m}}^{n}(3,3)\right]}^{2}+{\left[C_{b_{m}}^{n}(3,1)\right]}^{2}}\phi_{E}+\frac{C_{b_{m}}^{n}(3,1)C_{b_{m}}^{n}(1,3)}{{\left[C_{b_{m}}^{n}(3,3)\right]}^{2}+{\left[C_{b_{m}}^{n}(3,1)\right]}^{2}}\phi_{N}-\\ & \frac{C_{b_{m}}^{n}(3,1)C_{b_{m}}^{n}(3,2)}{{\left[C_{b_{m}}^{n}(3,3)\right]}^{2}+{\left[C_{b_{m}}^{n}(3,1)\right]}^{2}}\mu_{x}+\mu_{y}-\frac{C_{b_{m}}^{n}(3,2)C_{b_{m}}^{n}(3,3)}{{\left[C_{b_{m}}^{n}(3,3)\right]}^{2}+{\left[C_{b_{m}}^{n}(3,1)\right]}^{2}}\mu_{z} \end{array}$$

The corrected attitude measurement information could be obtained and its expression is as follows:

(34) $$\begin{array}{ll} \delta \psi & =\delta \psi^{\prime }-\frac{C_{b_{m}}^{n}(1,2)C_{b_{m}}^{n}(2,3)-C_{b_{m}}^{n}(1,2)C_{b_{m}}^{n}(2,2)}{{\left[C_{b_{m}}^{n}(1,2)\right]}^{2}+{\left[C_{b_{m}}^{n}(2,2)\right]}^{2}}(\sigma_{x}+\rho_{x})\\ & -\frac{C_{b_{m}}^{n}(1,1)C_{b_{m}}^{n}(2,2)-C_{b_{m}}^{n}(1,2)C_{b_{m}}^{n}(2,1)}{{\left[C_{b_{m}}^{n}(1,2)\right]}^{2}+{\left[C_{b_{m}}^{n}(2,2)\right]}^{2}}(\sigma_{z}+\rho_{z}) \end{array}$$

(35) $$\delta \theta =\delta \theta \prime -\frac{C_{b_{m}}^{n}(3,3)}{\sqrt{1-{\left[C_{b_{m}}^{n}(3,2)\right]}^{2}}}(\sigma_{x}+\rho_{y})+\frac{C_{b_{m}}^{n}(3,1)}{\sqrt{1-{\left[C_{b_{m}}^{n}(3,2)\right]}^{2}}}(\sigma_{z}+\rho_{z})$$

(36) $$\begin{array}{ll} \delta \gamma = & \delta \gamma^{\prime }+\frac{C_{b_{m}}^{n}(3,1)C_{b_{m}}^{n}(3,2)}{{\left[C_{b_{m}}^{n}(3,3)\right]}^{2}+{\left[C_{b_{m}}^{n}(3,1)\right]}^{2}}(\sigma_{x}+\rho_{x})-(\sigma_{y}+\rho_{y})\\ & +\frac{C_{b_{m}}^{n}(3,2)C_{b_{m}}^{n}(3,3)}{{\left[C_{b_{m}}^{n}(3,3)\right]}^{2}+{\left[C_{b_{m}}^{n}(3,1)\right]}^{2}}(\sigma_{z}+\rho_{z}) \end{array}$$

## 5. Results

### 5.1. System Composition

**Figure 9:**
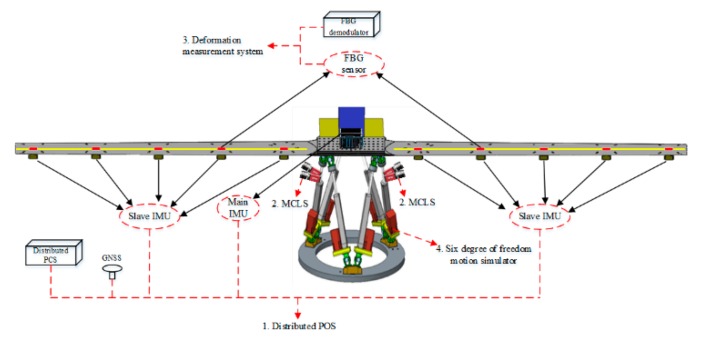
Overall composition diagram of the system.

The wing deformation test and verification system based on FBG is composed of a simulated wing structure, the FBG deformation measurement system, the DPOS, and six degree of freedom motion simulator. The simulation wing junction contains 10 sub-nodes on the left and right sides, and the whole wing structure is fixed by the mounting base. It is fixed on the six degree of freedom motion simulator, and the six degree of freedom motion simulator could simulate the typical motion environment of the aircraft, which includes the typical line motion and angular motion. The FBG deformation measurement system is composed of FBG sensors and a demodulator, in which the FBG sensor is installed on the upper surface and lower surface of the simulated wing structure. The surface corresponding position information is used to measure the wing deformation. The main IMU is mounted on the base of the analog wing structure, and the 10 sub-IMU are installed on the left and right side of the simulated wing structure, respectively. A schematic diagram of the system composition and installation is shown in Figure 9.

During the test, the dynamic deformation of the wing structure is simulated and the wing deformation is measured. The system provides simulated wing deformation environment under the simulated airborne environment; the FBG deformation measurement system transfers the high-precision wing deformation measurement to DPOS. Then the transfer alignment based on the FBG is completed using the high-precision main POS and the wing deformation measurement information, and the motion parameters of the sub-nodes is measured.

**Figure 10:**
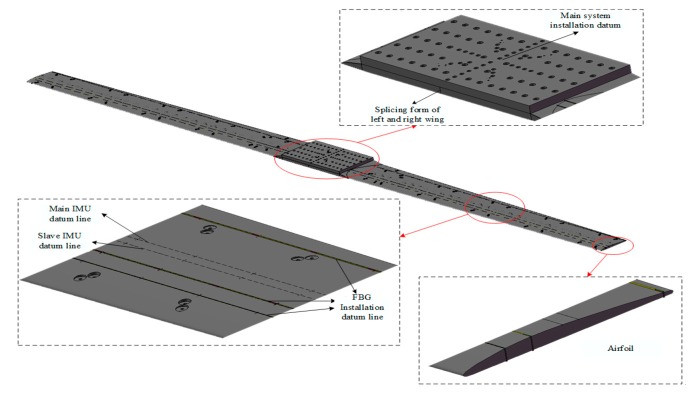
Simulation of wing structure.

The simulated wing structure is based on the real wing shape and structure, which is consistent with the real wing modal characteristics. The wing structure on both sides is connected in a stitching mode, which is fixed on the motion simulator by the upper pressure plate. Both sides of the wing used the classic flat convex airfoil CLARK-Y on the upper surface streamline and lower surface plane. The wing is 3 m long and the string length is 0.32 m; the other string length is 0.24 m. In addition, the main IMU is set on the surface of the upper pressure plate. The base is set up with five sub-IMUs on the lower surface of the wings of both sides, respectively. At the same time, the reference axis of the sub-IMUs and FBG are set on the upper and lower surfaces of the simulated wing. The specific parameters of the wing structure are shown in the Table 1 and the schematic diagram is shown in Figure 10.

FBG sensors are used to directly sense the strain changed at the mounting points. Its layout would directly affect the accuracy of deformation measurement of the simulated wing structure. FBG sensors should be installed as far as possible to enhance the signal-to-noise ratio of the signal. At the same time, the installation of the sensor could not affect the load and IMU installation.

**Figure 11:**
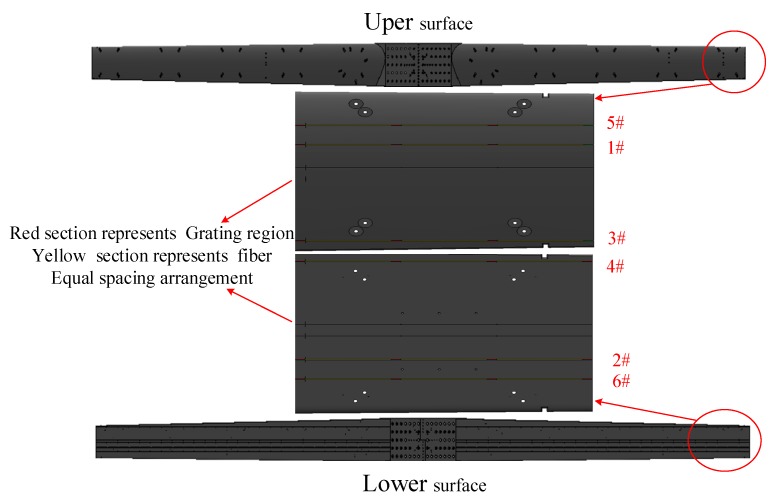
Schematic diagram of overall installation of fiber grating.

In order to reduce the influence of common measurement errors such as temperature and vibration on FBG sensors, the FBG sensors are installed at the corresponding position of the upper and lower surfaces of the wing structure, and the effect of the common error on the deformation measurement results is reduced through the reduction of the sensor signals on the upper and lower surfaces. In addition, the distance between the corresponding sensors should be increased as far as possible, so as to improve the division degree of the deformation signal. For the designed wing structure, six FBG sensors are installed on each wing, while there are three on the upper surface and three on the lower surface. Each fiber Bragg grating sensor contains 15 measuring points and also five sensors near the wing of the wing and the spacer 20 cm installation of 10 sensors. Through the arrangement of the FBG sensors, the network distribution measurement of the FBG sensors on the simulated wing structure is realized, as shows in Figure 11.

### 5.2. System Testing and Data Analysis

**Figure 12:**
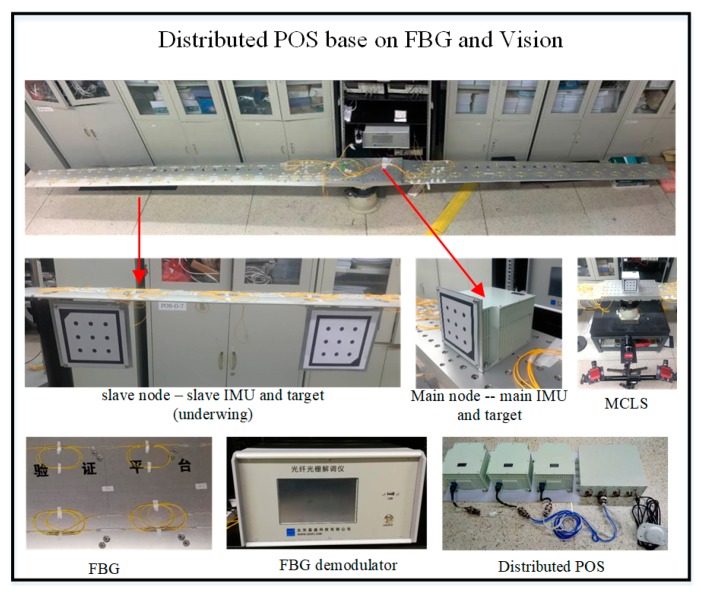
Composition of the test system.

The test equipment is shown in Figure 12. The FBG sensor is located on the upper and lower surfaces of the wing. The high-precision main IMU is fixed on the base, and the main IMU consists of three fiber optic gyroscopes, whose drift is 0.01 °/h and three accelerometers, whose bias is 10 μg. The low-precision sub-IMUs consists of MEMS-IMU. By continuously applying a weight on one side of the wing, the mooring wing is flexibly bent, while using a micrometer to continuously measure the displacement change for each deformation in the *Y*-axis direction.

#### 5.2.1. Measurement of Deformation Displacement of FBG

**Figure 13:**
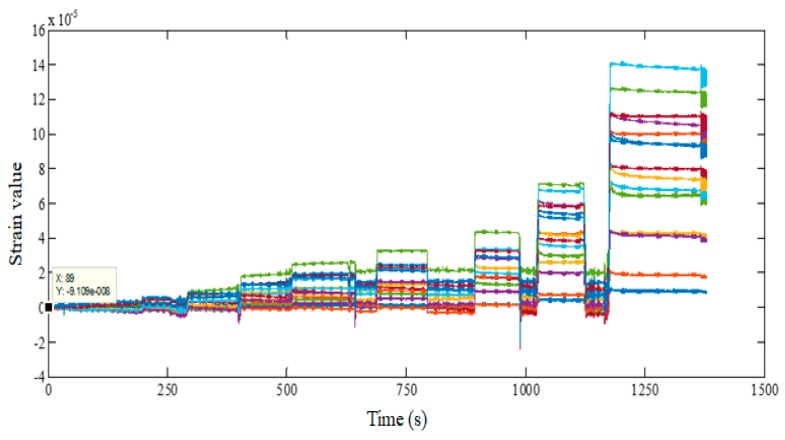
Change of strain with time at the point of fiber Bragg grating.

The wavelength data measured by FBG sensors on the upper and lower surface as well as the strain values of the measured points of the FBG sensors are calculated after the difference. Figure 13 shows the strain on the 15 measuring points of the flexible wing changed with time.

**Figure 14:**
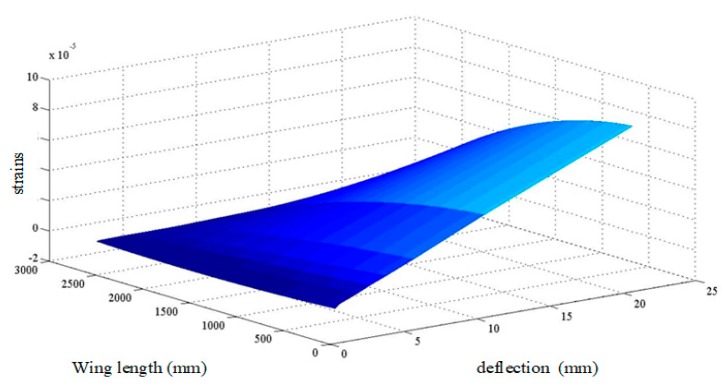
Fitting three-dimensional diagram of strain value.

According to the force condition of the flexible wing, the strain value is fitted two times. Figure 14 shows that the *X*-axis and the *Y*-axis are the end displacement values of the wing and the *Z*-axis is the strain value at each load weight.

**Figure 15:**
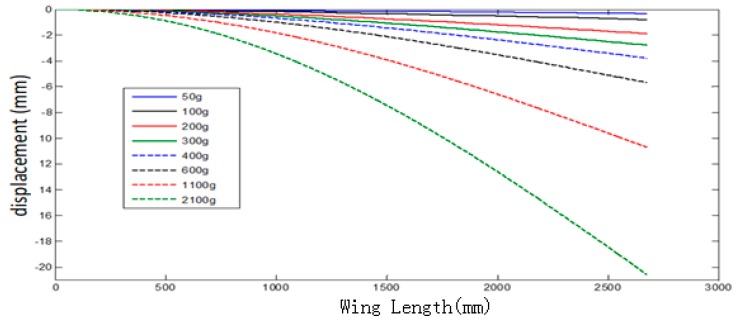
Under different loading conditions.

The strain curve after the fitting is plotted using second-order integration again, and the wing end displacement is measured by micrometer through loading 600, 1100 and 2100 g weight. In addition, the micrometer measurement is used as the reference data to calibrate the wing deflection curve obtained by the calculation, which is shown in Figure 15.

The calculated displacement of the wing tip is different from the displacement measured by the micrometer. The experimental results of measuring the deformation displacement for the simulated wing with FBG showed that the measuring accuracy of FBG sensor for one-dimensional linear displacement could reach 0.151 mm, as shown in Table 2.

#### 5.2.2. Transfer Alignment Test of Subsystem

**Figure 16:**
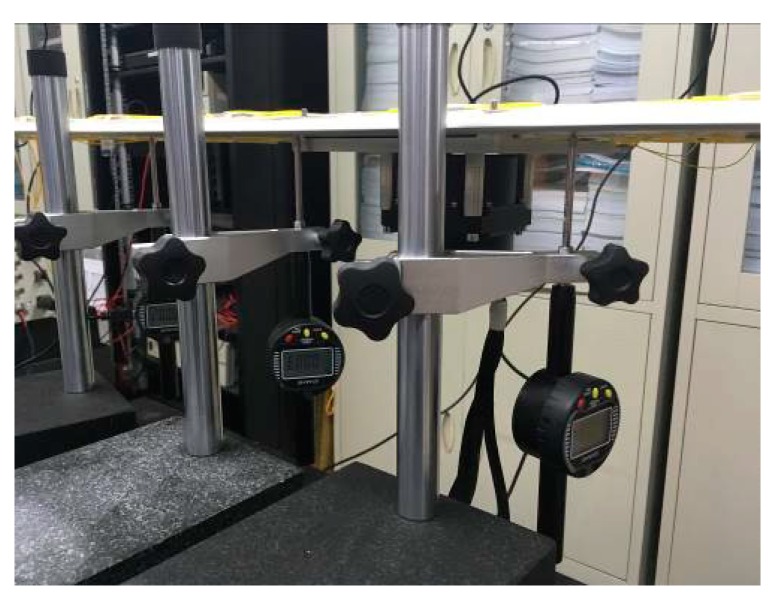
Micrometer working diagram.

The high-precision IMU of distributed POS is placed at the position of the main node, and two sub nodes are selected to place high-precision IMU and low-precision IMU on one side, that is slave system a1 and slave system a2. The two GPS antennas are connected with the connected IMU (eliminate the arm error between the GPS and the inertial navigation). At the same time, 2 μm are used to measure the position changes of two sub nodes respectively, as shown in Figure 16. Firstly, the wing frame is leveled and the test system is in the initial state. At this time, the distributed POS system starts to work and the fiber grating begins to preheat. After a certain time static test, the weight of the wing is added at the end of the wing to make the wing flexure and deform, which leads to the change of the sub nodes (the deformation is mainly in the direction of the vertical axis). When the wing is flexed and deformed, it will be tested again for a certain time. Two micrometers data m1 and m2 are read. At this point, transfer alignment of the sub-system a1 and the sub-system a2 are completed respectively by using the combined solution information of the master node and the deformation measurement information of the fiber Bragg grating; settlement results are b1 and b2. At the same time, the two subsystems and the differential GPS data are combined and solved afterwards; settlement results are c1 and c2.

**Figure 17:**
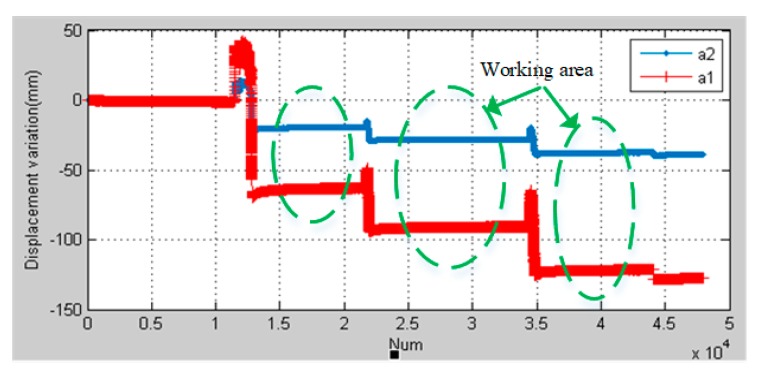
The system of a1 and a2 displacement change diagram.

The vertical displacement changes of two subsystems a1 and a2 after transfer alignment as shown in Figure 17. The work area after one stable deformation is selected, and the displacement changes $\Delta y_{FBG}$ of the sub-nodes are calculated using transfer alignment based on FBG, and the displacement changes $\Delta y_{DGPS}$ of the sub-nodes are calculated using DGPS, where $\Delta y_{FBG}=\left|b1-b2\right|$, $\Delta y_{DGPS}=\left|c1-c2\right|$. Comparison of the difference with the standard measured value $\Delta y_{M}$ from micrometers, where $\Delta y_{M}=\left|m1-m2\right|$. The results are shown in Table 3. It can be seen that the accuracy of DGPS is only to the centimeter level, so the millimeter level measurement cannot be realized. The measurement accuracy of the transfer alignment based on FBG can be less than 0.3 mm.

**Figure 18:**
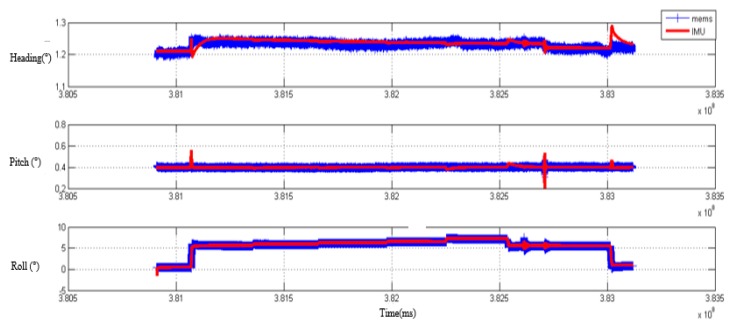
The attitude comparison from transfer alignment of MEMS and fiber IMU.

**Figure 19:**
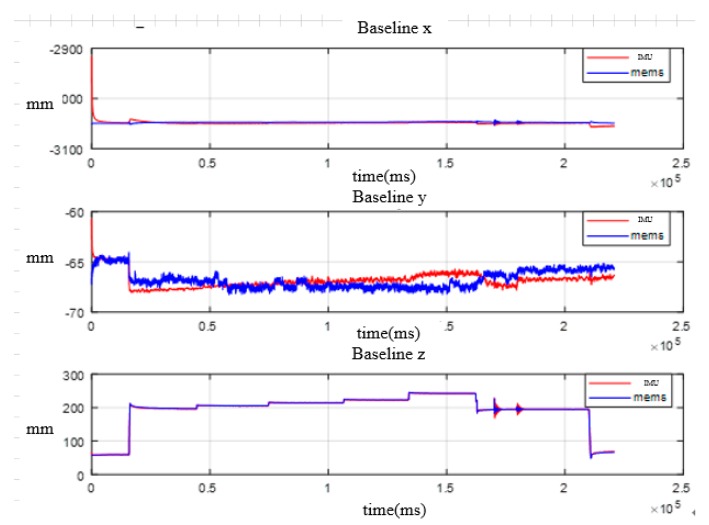
Comparison of wing deformation measurement.

First of all, the main system-based laser gyros and subsystem based on MEMS gyros conduct transfer alignment according to position and attitude matching method above, and obtain the attitude of the subsystem and the flexible baseline length of three dimension; second, the main system and the high-precision subsystem based on fiber gyros also conduct transfer alignment to obtain the attitude of the subsystem and the flexible baseline length of three dimensions. Finally, the two transfer alignment results are compared to verify the accuracy of transfer alignment. In the process, MEMS IMU and fiber IMU are rigidly connected at the end of the simulated wing. The comparison of the processed attitude results is shown in Figure 18 and Figure 19. The results from the fiber IMU are used as the reference, and the error statistics for attitude and baseline length are shown in Table 4 and Table 5.

## 6. Conclusions

A DPOS transfer alignment method based on FBG for flexible deformation compensation is studied. Firstly, the measurement error of matching parameter caused by flexible baseline is compensated by the deformation displacement and deformation angle measured using FBG sensors, then the transfer alignment model based on FBG assisted inertial measurement is established, and the position and attitude accuracy of the subsystem are improved by filtering estimation. Finally, the effectiveness of the transfer alignment method is verified by the DPOS ground demonstration system. In the future, some real field test will be conducted in the vehicle and during flight.

## Figures and Tables

**Table 1:** Simulation of wing structure parameters.

Parameter Type	Value
Single wing length	3000 mm
Airfoil length	2700 mm
Laminate length	600 mm
Laminate width	320 mm
Laminate thickness	25 mm
Chord length of the wing	320 mm
Chord length of the wingtip	240 mm
Root tip ratio	0.75
Maximum thickness	21 mm
Right wing weight	32.772 kg
Left wing weight	32.775 kg
Platen weight	12.852 kg
Installation interval between Main IMU and first sub IMU	600 mm
Installation interval between sub IMUs	560 mm
Wing and base fitting screw	M6
Platen and wing fitting screw	M8
Transition of the wing root	Quadrangular transition

**Table 2:** Measurement value of FBG deformation.

Num	Weights (g)	Micrometer Measured Value (mm)	FBG Measured Value (mm)	Deviation Value (mm)
1	50	−0.363	−0.295	0.068
2	100	−0.679	−0.773	0.094
3	200	−1.889	−1.874	0.015
4	300	−2.748	−2.769	0.021
5	400	−3.827	−3.775	0.052
6	600	−5.722	−5.671	0.051
7	1100	−10.537	−10.688	0.151
8	2100	−20.666	−20.558	0.108

**Table 3:** Comparison results of wing deformation measurement (mm).

Displacement Variation	Area 1	Area 2	Area 3
$\Delta y_{M}$	32.15	46.13	61.75
$\Delta y_{FBG}$	32.39	46.34	61.92
$\Delta y_{DGPS}$	53.57	64.82	80.11

**Table 4:** Transfer alignment between main POS and MEMS (°).

State	Heading MSE	Pitch RMSE	Roll RMSE
1	0.0089	0.0045	0.0790
2	0.0066	0.0025	0.1008
3	0.0058	0.0019	0.1007
4	0.0043	0.0023	0.0821
5	0.0038	0.0023	0.0827

**Table 5:** RMSE of wing deformation measurement (mm).

State	*x*	*y*	*z*
1	2.020	0.879	0.921
2	2.373	0.449	0.589
3	1.729	0.480	0.750
4	1.233	0.683	0.689
5	1.346	1.355	0.983
